# Induction of Diabetes Mellitus Using Alloxan in Sprague Dawley Rats

**DOI:** 10.7759/cureus.63359

**Published:** 2024-06-28

**Authors:** Jong-Min Kim

**Affiliations:** 1 Department of Animal Health, Cheongju University, Cheongju, KOR; 2 Transplantation Research Institute, Seoul National University Medical Research Center, Seoul National University, College of Medicine, Seoul, KOR

**Keywords:** induction rate, insulin treatment, rat, alloxan, diabetes mellitus

## Abstract

Purpose: The use of rodent models for diabetes, particularly with pancreatic islet transplantation, has been prevalent in various preclinical trials. The purpose of this study is to establish a diabetes mellitus (DM) model in Sprague Dawley (SD) rats using alloxan evaluated by assessing alloxan dosage, the induction rate of diabetes, and glucose stability through insulin treatment.

Methods: Over the course of 13 experimental rounds, diabetes was induced in 86 SD rats using alloxan at concentrations of 200 mg/kg (16 rats) or 150 mg/kg (70 rats). Various parameters, including diabetes induction rates, average insulin doses, extent of weight loss, and adverse effects such as diabetic ketoacidosis (DKA), were measured.

Results: The administration of 200 mg/kg of alloxan in rats resulted in severe diabetes induction, leading to DKA in three individuals, despite daily insulin glargine administration, DKA prevention was unsuccessful. The stability of alloxan decreases over time, especially when refrigeration is compromised during weighing. In the group treated with 150 mg/kg of alloxan, the diabetes induction rate was 83%. The average insulin dose was 2.21 units/kg/day. In contrast, the group treated with 200 mg/kg of alloxan exhibited a diabetes induction rate of 81% with a statistically significant higher average insulin requirement at 7.58 units/kg/day compared to 150 mg/kg of alloxan.

Conclusion: Inducing diabetes in rats with 150 mg/kg of alloxan is considered more suitable for creating a diabetes model for xenogeneic islet transplantation compared to using 200 mg/kg of alloxan. This is due to fewer complications related to DKA or hyperglycemia and reduced need for exogenous insulin treatment.

## Introduction

Diabetes mellitus (DM) represents a group of physiological dysfunctions characterized by hyperglycemia, resulting from insulin resistance (as seen in type 2 diabetes mellitus-T2DM), inadequate insulin secretion/production, or excessive glucagon secretion (in type 1 diabetes mellitus-T1DM). Type 1 diabetes is a chronic, progressive autoimmune disease affecting approximately 1% of the population in the developed world. In contrast, type 2 diabetes is primarily caused by insulin resistance coupled with reduced insulin output, affecting approximately 8.5% of the adult population [1]. For patients with poorly controlled diabetes especially type 1 DM, despite insulin therapy, allogeneic islet transplantation with immunosuppressant has been undergoing clinical trial [2]. However, while allogeneic islets are preferable due to lower immune responses compared to xenogeneic islets, their availability of supply is limited. To overcome this challenge, research is underway to use xenogeneic pancreatic islets, particularly from pigs, which offer an infinite supply despite the potential for a greater immune response compared to allogeneic islets [3]. This approach aims to advance diabetes treatment. Xenogeneic porcine pancreatic islet transplantation, particularly when performed via the portal vein in non-human primate (NHP) models, often encounters strong cellular and humoral rejection responses, necessitating potent immunosuppressants [4]. To overcome these challenges, encapsulated islet transplantation has been investigated as an alternative strategy for diabetes management, aiming to shield transplanted islets from the host immune response [5].

Before attempting clinical trials with encapsulated porcine xenogeneic islets incorporating state-of-the-art technologies, it is imperative to validate their efficacy in the diabetic NHP model [6]. However, considering the high costs associated with non-clinical trials in the diabetic NHP model, it is essential to precede efficacy assessments in a diabetic rat model. Therefore, the objective of this study is to establish a diabetes model in rats using alloxan, known for destroying beta cells by chemical compounds, to evaluate the dose of alloxan, the induction rate of DM, and the stability of blood glucose with insulin treatment.

## Materials and methods

Overview

DM was induced by intraperitoneal injection of alloxan in 86 SD rats (Table 1). They were expected to be transplanted with encapsulated islets within 3 to 7 days. A total of 13 experimental rounds were conducted using 86 SD rats. In experimental rounds 1-3, 16 SD rats received a dose of 200 mg/kg of alloxan one week before transplantation. For experimental rounds 4-13, 70 SD rats received a dose of 150 mg/kg of alloxan three days before transplantation. Each experimental round occurred approximately one month apart. Experimental rounds 1-11 and experimental rounds 12-13 utilized different batches of alloxan. Until experimental round 11, alloxan was taken out of the refrigerator for 10 minutes at room temperature when measuring its weight. Starting from experimental round 12, to maintain the heat stability of alloxan, the weighing procedure was also conducted with the reagent bottle kept on ice to ensure refrigeration. The animal experiments were approved by the Institutional Animal Care and Use Committee (IACUC) of the Biomedical Research Institute at the Seoul National University Hospital (AAALAC accredited facility; IACUC number: 23-0236).

Induction of DM by alloxan

Alloxan monohydrate (Sigma-Aldrich, St Louis, MO, USA) was dissolved in normal saline to achieve a concentration of 5% (W/V) before injection of each rat. For example, the weight of the rats was measured one day before the alloxan administration. If the dosage was 150 mg/kg and the rats weighed between 300 and 350 g, 60 mg of alloxan (corresponding to a 400 g rat) was placed in Eppendorf tubes and kept refrigerated. On the day of administration, to prepare a 5% (W/V) solution, 1.2 ml of saline was added to the alloxan in the animal facility. Then, 1.05 ml of the solution was administered intraperitoneally to a 350 g rat, and 0.9 ml was administered to a 300 g rat. After alloxan injection, an oral solution of 10% sugar in tap water was provided via a water bottle ad libitum for one day to prevent hypoglycemic shock.

Insulin treatment procedure, daily insulin requirement, and the complications of insulin treatment in diabetic SD rats

**Figure 1:**
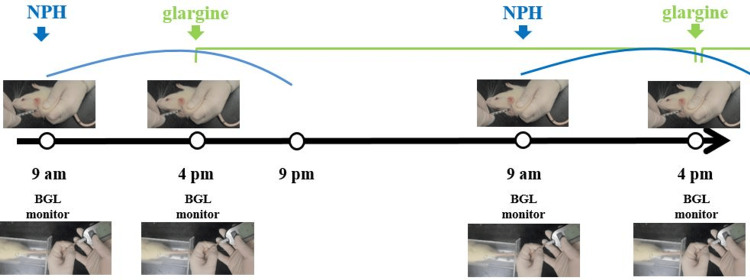
Insulin treatment protocol in alloxan-induced rat DM model. The rats were fed a diet ad libitum. The blood glucose level (BGL) at 9 am in the morning is dependent on the dose of glargine that had been injected at 4 pm on the previous day. BGL at 4 pm is mainly influenced by NPH that had been injected at 9 am. Blue (NPH) curves or green (glargine) lines indicate the estimated pharmacokinetics of those insulin formulations. (Image credit: Jong Min Kim) NPH: neutral protamine Hagedorn

The rats were fed a gamma ray-irradiated rodent diet ad libitum (lab rodent chow 95135, Cargill Agri Purina Korea Inc., Pyungtaek Seongnam Kyunggi-do, Korea). Body weight was measured daily following alloxan administration. Blood glucose concentrations were measured using a small electrode-type blood glucose meter (Accu-Chek™, Roche Diagnostics, Seoul, Korea). Complete diabetes was confirmed by persistent hyperglycemia over 300 mg/dl of blood glucose level. After confirming complete DM induction, blood glucose level (BGL) was checked at least two times per day. To regulate BGLs, in experimental round 1, glargine was administered once daily. From experimental round 2 onwards, neutral protamine Hagedorn (NPH) was administered in the morning, and glargine was administered in the afternoon. The desired target value of the fasting BGL was approximately 80 - 150 mg/dl in the diabetic rats (Figure 1).

For the starting dose, 0.5 IU insulin per head based on the BGL was injected. Subsequently, insulin dose adjustments which have been modified from the method used in streptozotocin (STZ)-induced diabetic rhesus macaque [7] were applied to maintain target BGL. Daily insulin requirement was measured in successfully DM-induced SD rats that are expected to be transplanted with encapsulated islets within 3 to 7 days after DM induction by alloxan. Complications of insulin treatment in diabetic SD rats were observed. DKA was defined as over 600 mg/dl of BGL during over one day, ketone positive in urine using urine dipstick, acidosis in venous gas analysis, and over 15% weight loss (Table 1).

**Table 1:** Insulin dose adjustments by blood glucose levels Insulin dose adjustment involves determining the insulin dosage based on the measured blood glucose levels (BGL). For instance, if the afternoon BGL measures between 80-100 mg/dl, the next morning’s dose of NPH insulin is reduced by 0.25 IU compared to the morning dose administered on the current day. If the afternoon BGL measures between 400 and 600 mg/dl, the next morning’s dose of NPH insulin is increased by 0.75 IU compared to the dose administered on the current day. However, if the afternoon BGL measures <80 mg/dl, no glargine insulin is administered in the afternoon.

BGL (mg/dl)	Insulin Adjustment Dose per head
<80	No insulin injection
80-100	-0.25 IU
100-200	0 IU
300-400	0.5 IU
400-600	0.75 IU
>600	1.0 IU

Statistical analysis

The average insulin dose between the groups receiving 200 mg/kg and 150 mg/kg of alloxan was analyzed using the t-test (nonparametric, Mann-Whitney) method. Weight loss was expressed as mean ± standard deviation. Statistical analysis for weight loss across experimental rounds was conducted using one-way ANOVA (Kruskal-Wallis, Dunn’s Multiple Comparison Test). The comparison of weight loss between the groups receiving 200 mg/kg and 150 mg/kg of alloxan was performed using the t-test (nonparametric, Mann-Whitney) method. GraphPad Prism software was utilized for these analyses, and a p-value < 0.05 was considered statistically significant.

## Results

Administering 200 mg/kg of alloxan in rats induced severe diabetes, and once daily administration of glargine was insufficient to prevent diabetic ketoacidosis (DKA).

In experimental round 1, using 200 mg/kg of alloxan, three out of six rats developed DKA (Table 2).

**Table 2:** Overview of alloxan-induced DM in Sprague Dawley rats Experimental rounds 1-11 and experimental rounds 12-13 used different batches of alloxan. Each experimental round occurred at approximately one-month intervals. Experimental rounds 1-3 induced diabetes one week before transplantation. Diabetes was induced three days before transplantation from experimental round 4. In experimental round 1, high BGL was maintained with once-daily glargine administration, and from experimental round 2, high BGL was regulated with NPH in the morning and glargine in the afternoon. There was a statistically significant difference in the average insulin dose when comparing 200 mg/kg of alloxan to 150 mg/kg of alloxan as determined by a t-test (nonparametric, Mann-Whitney). A p-value < 0.05 was considered statistically significant. N/A and - indicate not applicable and data not observed, respectively. In the experimental rounds following the induction of diabetes in rats by alloxan, there was no statistically significant difference in weight loss when comparing different experimental rounds (analyzed using one-way ANOVA, Kruskal-Wallis, and Dunn’s Multiple Comparison Test). Furthermore, when comparing weight loss after diabetes induction in rats based on the concentration of alloxan administered, there was also no statistically significant difference between the groups treated with 200 mg/kg and 150 mg/kg of alloxan (analyzed using t-test with nonparametric methods such as Mann-Whitney). IP: intraperitoneal injection; DKA: diabetic ketoacidosis; SD: standard deviation.

Experimental round	Number of rats used	Alloxan (mg/kg, IP)	Induction rate of diabetes mellitus (%)	Morbidity number of DKA (expired day)	Average insulin dose (IU/kg/day)	Weight loss (%, mean ± SD)
1	6	200	83	3 (D4, D5, D6)	10.3 (p-value < 0.0001 vs 150 mg/kg of alloxan)	15 ± 12
2	6	200	83	-	7.0 (p-value < 0.0001 vs 150 mg/kg of alloxan)	10 ± 2
3	4	200	75	-	5.4 (p-value < 0.0001 vs 150 mg/kg of alloxan)	8 ± 2
4	3	150	100	-	2.6	15 ± 4
5	3	150	66	-	3.3	14 ± 1
6	4	150	75	-	4.7	4 ± 6
7	9	150	78	-	3.1	10 ± 5
8	7	150	86	-	1.6	11 ± 5
9	8	150	75	-	0.6	4 ± 11
10	9	150	56	-	1.6	12 ± 2
11	9	150	22	-	1.5	15 ± 3
12	9	150	88	-	1.5	11 ± 3
13	9	150	88	-	1.7	11 ± 4
Total	86	N/A	74	N/A	N/A	N/A

Despite attempting to lower hyperglycemia by administering glargine once daily and gradually increasing the daily insulin dose (average insulin dose; 10.3 IU/kg/day), the severity of DKA led to the death of three rats on days 4, 5, and 6. It was confirmed that once-daily glargine administration was insufficient for glucose control. Therefore, in experimental rounds 2 and 3, blood glucose management was achieved by administering NPH in the morning and glargine in the afternoon (average insulin dose for round 2; 7.0 IU/kg/day, average insulin dose for round 3; 5.4 IU/kg/day). Until the transplantation, adequate diabetes control was maintained without the occurrence of DKA. However, considering the potential side effects leading to DKA with the administration of 200 mg/kg of alloxan, the decision was made to reduce the alloxan dosage to 150 mg/kg. Furthermore, managing blood glucose for a minimum of 3-4 days before encapsulated pancreatic islet transplantation posed a significant challenge. Observing that diabetes was reliably induced when alloxan was administered on day 2 (mostly with BGLs exceeding 300 mg/dl on day 1), starting from experimental round 4, diabetes induction was shifted to 3 days before transplantation to alleviate the efforts in blood glucose management and insulin administration.

The stability of alloxan in the reagent bottle further diminished if refrigeration is compromised, especially during weighing at room temperature. Alloxan should be refrigerated for stability. We withdrew the required amount of alloxan from refrigerated storage for experimental rounds 1-11, allowing it to sit at room temperature for 10 minutes before returning it to refrigeration. The withdrawn alloxan was kept on ice and then diluted in saline upon arrival in the animal facility and administered into the rat peritoneal cavity. However, starting from experimental rounds 10 and 11, the potency of alloxan declined during the process of measuring its weight at room temperature, resulting in a reduction in the diabetes induction rates to 56% and 22%, respectively. Considering the diabetes induction rates, exposure to room temperature for 10 minutes during each experimental round appeared to maintain the potency of alloxan up to round 9, but thereafter, a significant decrease in potency was observed. Due to the decreased diabetes induction rates observed in experimental rounds 10 and 11, we introduced a new batch of alloxan. To address refrigeration issue, we modified the protocol by placing the alloxan bottle on ice during weight measurements to maintain refrigeration. In experimental rounds 12 and 13, utilizing the new batch, the diabetes induction rate increased to 88%. While the initial batch of alloxan did not show issues with diabetes induction rates until experimental round 9, further experimentation with the new batch is warranted. However, it is essential to note that alloxan’s known susceptibility to heat instability emphasizes the importance of careful attention to all procedures until the administration of alloxan.

The administration of 150 mg/kg of alloxan-induced diabetes with fewer side effects. In rats from experimental rounds 4-9 (34 rats) and experimental rounds 12-13 (18 rats), a diabetes induction rate of 83% was achieved (9 failures to induce, 43 successful inductions). This is comparable to the diabetes induction rate observed in the initial rounds 1-3 with 16 rats, which was 81% (3 failures to induce, 13 successful inductions). Statistically, there is no significant difference in the diabetes induction rate and the extent of weight loss between the groups receiving 200 mg/kg and 150 mg/kg of alloxan (Table 1). However, 150 mg/kg of alloxan did not induce DKA. This can be attributed to the administration of insulin twice a day (NPH and glargine), as observed in experimental rounds 2 and 3. Nonetheless, in experimental rounds 4-13 with 150 mg/kg of alloxan, the average insulin dose was 2.21 IU/kg/day. In contrast, experimental rounds 1-3 with 200 mg/kg of alloxan exhibited a statistically significant higher insulin requirement at 7.58 IU/kg/day (Table 1). This suggests that beta cells in rat pancreas are more severely destroyed by 200 mg/kg of alloxan, leading to a significant difference in insulin requirements. Consequently, the lower insulin requirement in the 150 mg/kg group may result in fewer side effects associated with hyperglycemia.

## Discussion

The initial dosage of alloxan at 200 mg/kg was chosen based on the study by Federiuk et al. [8], which reported a 70% incidence and 10% mortality when 200 mg/kg of alloxan was administered. However, as mentioned in the results section, administering 200 mg/kg of alloxan-induced severe diabetes leads to DKA with only a once-daily glargine injection. It was necessary to administer both NPH and glargine twice daily to reduce hyperglycemia effectively. Moreover, the required transplanted islet mass for achieving insulin independence post-xenogeneic islet transplantation was significantly high (data not shown). Therefore, we concluded that reducing the severity of diabetes induction would facilitate achieving insulin independence with a lower transplanted islet mass. Consequently, we decided to decrease the alloxan dosage to 150 mg/kg. Even at 150 mg/kg, while hyperglycemia remained stable, it did not completely eliminate the need for exogenous insulin administered twice daily (morning and evening). Hence, we determined that 150 mg/kg of alloxan was adequate to induce diabetes in rats and to produce reliable results post-xenogeneic islet transplantation.

Alloxan and STZ are commonly employed chemical compounds to induce diabetes in animal DM models. The mechanisms underlying their diabetes-inducing effects involve the selective uptake of cytotoxic glucose analogs through the GLUT2 glucose transporter in beta cells. This leads to beta cell necrosis, with alloxan inducing reactive oxygen species and STZ causing DNA alkylation. The resultant insulin deficiency contributes to the development of diabetes in animal DM models [9]. However, alloxan offers the advantage of being approximately 70 times more cost-effective than STZ (Currently, the price of alloxan sold by Sigma-Aldrich is approximately $6.75 per g, whereas STZ is approximately $511 per gram.). While STZ requires storage at -20°C, alloxan can be stored in a regular refrigerator. One drawback of alloxan is its reduced stability when dissolved compared to STZ. Alloxan has a short half-life of 1.5 minutes at pH 7.4 and 37°C, necessitating immediate administration after dilution. In contrast, STZ remains stable for up to 1 hour under similar conditions after dissolution. Therefore, when using alloxan, the induction rate can be significantly influenced when dissolving and administering to a large number of animals simultaneously. Using freshly dissolved alloxan for each administration mitigates the impact on the induction rate of DM [10]. In this study, recognizing the drawbacks of alloxan, we took precautions by dissolving alloxan for each animal individually before immediate administration, resulting in an induction rate of over 80%. However, upon reviewing experimental rounds 10 and 11, it is evident that exposing alloxan to room temperature for approximately 10 minutes during the weighing process can compromise its stability. Therefore, it is advisable to maintain alloxan on ice when measuring its weight each time to ensure stability.

In a study, it was demonstrated that the induction rates of diabetes in rats were similar when administering appropriate concentrations of two drugs (140 mg/kg of alloxan; 80% vs 40 mg/kg of STZ; 79%) [11]. In this study as well, it is inferred that similar results were observed, with a diabetes induction rate of 83% for 150 mg/kg of alloxan and 81% for 200 mg/kg of alloxan. In comparing the diabetes induction rates of the DM rat model using alloxan, inbred rats are typically employed [11-13]. However, for this experiment, outbred SD rats were used, introducing genetic diversity. While this diversity in genetic background may lead to variations in the diabetes induction rate with alloxan, our experimental objective was to successfully establish encapsulated porcine xenogeneic islets in a wild-type rat DM model, with the ultimate goal of transitioning to a wild-type NHP DM model. Therefore, outbred rats were chosen for this specific purpose.

In rat DM model induced by STZ or alloxan, there are severe DM models (characterized by the marked deficiency or absence of endogenous insulin and C-peptide, and a tendency toward ketosis when exogenous insulin administration is insufficient to prevent beta-oxidation of fatty acids) or moderate DM model (characterized by more stable hyperglycemia and absent or very low ketone concentration with or without exogenous insulin administration), depending on the purpose [14]. For a team studying islet transplantation, a severe DM model is necessary because they want the transplanted animal’s blood glucose to be managed by the exogenously transplanted islets, not by endogenous insulin. On the other hand, a team researching automated insulin delivery requires a moderate DM model, where some endogenous insulin secretion is maintained, making blood glucose control easier and requiring less rigor in terms of automated insulin delivery [14]. Therefore, the amount of remaining endogenous islets appears to be closely related to islet regeneration in the chemically induced DM model. The mechanisms of islet regeneration after STZ injury are divided into intra-islet (beta cell self-replication or neogenesis from conversion of alpha to beta cells) and extra-islet (neogenesis from ductal progenitors) [15]. Intra-islet regeneration has been demonstrated in mice using the partial pancreatectomy model, as well as the pancreatic duct ligation and alloxan administration model [16,17]. This suggests that in the case of a moderate DM model rather than a severe DM model, the remaining beta cells may have the potential to regenerate. Therefore, in a DM induced by STZ or alloxan where exogenous insulin is not administered, intra-islet regeneration might be possible. For instance, one study reported that out of 27 SD rats administered 120 mg/kg of alloxan intraperitoneally, 3 died, while 24 developed diabetes but showed self-recovery starting from 2 weeks after induction. In this study, the 24 rats that developed diabetes did not receive insulin treatment and survived for 20 days without mortality. Immunohistochemical analysis revealed the presence of insulin-positive islets in the pancreas, suggesting a significant number of remaining islets, which are likely to have regenerated [18]. In another study, alloxan was administered intraperitoneally to groups of five SD rats at doses of 120, 150, and 180 mg/kg. The highest mortality rate was observed at 180 mg/kg, the highest induction rate at 150 mg/kg, and the highest induction failure rate at 120 mg/kg. In all three dose groups, four rats exhibited self-recovery from diabetes within 2-4 weeks [19]. However, these groups also did not administer insulin, and the animals survived for four weeks, indicating a moderate DM model, which suggests the possibility of intra-islet regeneration. However, in this experiment, the severe DM model induced DKA even though in the exogenous insulin treatment, making intra-islet regeneration of beta cells unlikely. Additionally, although alpha-to-beta cell conversion is considered possible, it was not observed histopathologically over a 21-day period in a high-dose STZ model (60 mg/kg) in rats, instead, this research team histopathologically confirmed that regeneration primarily occurred from centroacinar exocrine cells [20]. However, from a functional perspective, BGLs remained high after STZ administration, and there was no significant increase in blood insulin levels. Therefore, the regenerated insulin-positive cells observed in immunostaining did not play a significant role in aiding glucose metabolism. Although this study mainly discussed the induction and management of diabetes up to transplantation, 54 diabetic rats underwent encapsulated islet transplantation. Among the cases observed for over 20 days, excluding the six rats for which encapsulated islet transplantation was successful, no decrease in insulin dosage was observed over 20 days, suggesting a decreased likelihood of self-recovery at doses of alloxan 150 mg/kg or 200 mg/kg (data not shown). While there is much debate regarding self-recovery in rats induced with alloxan or STZ, this study did not find evidence supporting it, indicating the need for further research.

A limitation of this study is that the inability to measure rat C-peptide levels for both 150 mg/kg and 200 mg/kg of alloxan-treated rats prevents the assessment of residual islet function, making it challenging to clearly compare the differences in insulin requirements between the two groups. It could be considered a weak point of this experiment that the determination of lower side effects for 150 mg/kg of alloxan compared to 200 mg/kg of alloxan without measuring the rat’s remaining C-peptide level. However, since 150 mg/kg of alloxan showed comparable induction rates, lower insulin requirements, and no adverse effects like DKA when compared to 200 mg/kg of alloxan, it is considered a more stable concentration for inducing diabetes and managing hyperglycemia with insulin.

## Conclusions

In conclusion, due to the thermal instability of alloxan, it is important to maintain alloxan in a refrigerated state at all times. Alloxan is cheaper compared to STZ, and when compared to the results of other research groups inducing diabetes in rats using STZ, this study showed no significant difference in the diabetes induction rate in rats. In this study, when diabetes was induced with alloxan, approximately 80% of the rats showed diabetes induction within 1-2 days. Treatment with NPH and glargine insulin at a dose of 150 mg/kg of alloxan-induced diabetes required less insulin administration than 200 mg/kg of alloxan. This suggests that effective glycemic control can be achieved with a lower amount of insulin administration with 150 mg/kg of alloxan. Therefore, 150 mg/kg of alloxan to induce DM in rats is deemed to provide a more stable model for evaluating the efficacy of encapsulated porcine xenogeneic islets compared to 200 mg/kg of alloxan.
